# Lung microbiome of stable and exacerbated COPD patients in Tshwane, South Africa

**DOI:** 10.1038/s41598-021-99127-w

**Published:** 2021-10-05

**Authors:** T. Goolam Mahomed, R. P. H. Peters, M. Allam, A. Ismail, S. Mtshali, A. Goolam Mahomed, V. Ueckermann, M. M. Kock, M. M. Ehlers

**Affiliations:** 1 Department of Medical Microbiology, University of Pretoria, Pretoria, South Africa; 2 Foundation for Professional Development, Research Unit, East London, South Africa; 3 National Institute for Communicable Diseases, National Health Laboratory Service, Johannesburg, South Africa; 4 Louis Pasteur Private Hospital, Pretoria, South Africa; 5 Department of Internal Medicine, University of Pretoria, Pretoria, South Africa; 6 Department of Medical Microbiology, Tshwane Academic Division, National Health Laboratory Service, Johannesburg, South Africa

**Keywords:** Bioinformatics, Microbiome, Chronic obstructive pulmonary disease, Metagenomics

## Abstract

Chronic obstructive pulmonary disease (COPD) is characterised by the occurrence of exacerbations triggered by infections. The aim of this study was to determine the composition of the lung microbiome and lung virome in patients with COPD in an African setting and to compare their composition between the stable and exacerbated states. Twenty-four adult COPD patients were recruited from three hospitals. Sputum was collected and bacterial DNA was extracted. Targeted metagenomics was performed to determine the microbiome composition. Viral DNA and RNA were extracted from selected samples followed by cDNA conversion. Shotgun metagenomics sequencing was performed on pooled DNA and RNA. The most abundant phyla across all samples were *Firmicutes* and *Proteobacteria*. The following genera were most prevalent: *Haemophilus* and *Streptococcus*. There were no considerable differences for alpha and beta diversity measures between the disease states. However, a difference in the abundances between disease states was observed for: (i) *Serratia* (3% lower abundance in exacerbated state), (ii) *Granulicatella* (2.2% higher abundance in exacerbated state), (iii) *Haemophilus* (5.7% higher abundance in exacerbated state) and (iv) *Veillonella* (2.5% higher abundance in exacerbated state)*.* Virome analysis showed a high abundance of the BeAn 58058 virus, a member of the *Poxviridae* family, in all six samples (90% to 94%)*.* This study is among the first to report lung microbiome composition in COPD patients from Africa. In this small sample set, no differences in alpha or beta diversity between stable and exacerbated disease state was observed, but an unexpectedly high frequency of BeAn 58058 virus was observed. These observations highlight the need for further research of the lung microbiome of COPD patients in African settings.

## Introduction

Chronic obstructive pulmonary disease (COPD) is a progressive lung disease that results in progressive airflow limitation (i.e. obstruction)^1,2^. COPD is one of the world’s leading causes of death and was projected to be the third leading cause of death in 2020^3^. Symptoms of COPD include a chronic cough, dyspnoea and sputum production^4,5^. These symptoms affect the quality of life of the individual suffering from this disease^6^. There is limited data about the prevalence of COPD in the African continent; the last reported prevalence data on COPD in South Africa was in 2005 (19% in men and women over 40 years of age)^7–10^. This disease has been linked to smoking, exposure to occupational dust (e.g. working in a mine), burning of biomass and fossil fuels, previous tuberculosis infection and to HIV; all of these risk factors are highly prevalent in South Africa^10^.

Exacerbation of airway inflammation and associated symptoms is another factor that affects the quality of life for these individuals^10^. Patients suffering from COPD often move between a stable state of disease (where symptoms are absent to mild) to an exacerbated state of disease (defined as worsening of symptoms, respiratory and/or non-respiratory and over the course of the disease, as the lung damage due to COPD progresses, the frequency of these exacerbations increases^11–13^. Exacerbations are triggered by environmental pollutants, may have an unknown cause or by infection with bacteria and/or viruses^14^. Bacterial and viral infections account for between 30 to 50% of all exacerbations^15^. However, bacteria have been detected in the stable state of disease as well and the association between these microorganisms and disease is unclear^16,17^.

To better understand the role of microorganisms in COPD disease, the use of next-generation sequencing (NGS) can be employed to study the microbiome (defined as the genetic material of the microorganism in the community)^18^. NGS is high-throughput, parallel sequencing technology which has been used to sequence whole genomes of bacteria and viruses, perform transcriptomics (studying the complete set of RNA transcripts produced by the genomes) and to study the microbiome/metagenome^19,20^. The advantage of NGS over culturing and other molecular methods is that it can detect unculturable bacteria and provide information regarding the diversity, composition and functional roles of members of the microbiome^21,22^. An important drawback is that the cost of sequencing is still relatively high, especially in the African continent^23^. The NGS technology can be employed in one of two ways: (i) using a targeted approach or (ii) using a metagenomic approach^24,25^.

The targeted approach is commonly used to study the microbiome and is employed by targeting the 16S rRNA gene^26,27^. This gene is useful for studying the bacterial microbiome as it is universally present and conserved within all bacteria^28–30^. Studying the virome, i.e. viral component of the microbiome is more challenging as (i) most viruses are difficult to culture, (ii) there is no consensus sequence to study viruses and (iii) viruses are diverse and may be ssDNA, ssRNA, dsDNA or dsRNA^31–33^. By using shotgun metagenomics (i.e. random sequencing of the DNA from the microbial community) along with cDNA synthesis to study the virome, these challenges can be overcome^34–36^.

In South Africa, there is no data on the composition of the lung microbiome in COPD patients.

Previous studies on the lung microbiome of COPD patients were conducted in Europe and the USA^37–39^. Furthermore, there have been limited studies on the lung virome in COPD^40,41^. It is important to study not only the microbiome in the African continent, in countries such as South Africa but also the virome as local environmental conditions e.g. climate and clinical co-morbidities, e.g. HIV and tuberculosis infection (both of which are highly prevalent in sub-Saharan Africa) have the potential to affect the microbiome. Therefore, the aim of this study was to determine the composition of the lung microbiome and the lung virome in the sputum of COPD patients from South Africa and to compare their composition between stable and exacerbated states of disease.

## Methods

### Study setting and patient recruitment criteria

COPD patients admitted to or attending clinics (for scheduled check-ups) at one of three hospitals (one academic, one district and one private) in the Tshwane Health district, South Africa were invited to participate in the study. Written informed consent was obtained from all participants if the inclusion and exclusion criteria were met (Supplementary materials Table S1). Participants were classified as either in the stable or in the exacerbated state based on the definition by Vogelmeier et al*.* (2017). Ethical approval was granted from the Research Ethics Committee, Faculty of Health Sciences, University of Pretoria (REC no: 237/2017).

### Extraction of DNA and RNA and cDNA synthesis

Spontaneously expectorated sputum specimens were collected from participants at a single time point, transported on ice and stored at − 80 °C (Innova U535 Upright, Eppendorf, Germany) until batch processing could occur (no preservation medium was used). The sputum specimens were treated with an equal volume of 0.1% dithiothreitol (DTT) (Roche Diagnostics, Switzerland) to reduce sputum viscosity and homogenised for 30 s (Vortex-Genie^®^ 2; Scientific Industries Inc., USA)^42–44^. The samples were split into three aliquots for: (i) bacterial DNA extraction (aliquot 1), (ii) viral DNA and RNA extraction (aliquot 2) and (iii) storage at − 80 °C (aliquot 3, for future processing and/or studies) (Innova U535 Upright, Eppendorf, Germany).

The bacterial extraction aliquot was centrifuged (Spectrafuge™ 24D, Labnet International Inc., USA) at 4000×*g* for 30 min before extraction. Bacterial DNA was extracted using the Isolate II Genomic DNA Kit (Bioline, UK). The manufacturer’s instructions (protocol 9.2) were followed with the addition of 10 mg/mL lysozyme (Sigma-Aldrich, USA), 3 U/µL lysostaphin (Sigma-Aldrich, USA) and 6.75 µL of 10 U/µL mutanolysin (Sigma-Aldrich, USA) to the hard-to-lyse buffer [20 mM Tris (Sigma-Aldrich, USA) pH 8.0; 1% Triton X-100 (Amresco, USA); 2 mM EDTA (Sigma-Aldrich, USA)].

The viral DNA and RNA aliquot was treated with DNase I to remove host (human) DNA [10 U/mL TURBO™ DNase (Ambion, USA)] at 37 °C for 30 min (AccuBlock™ Digital Dry Bath, Labnet International Inc., USA), followed by inactivation with 15 mM ethylenediaminetetraacetic acid (EDTA) (Sigma-Aldrich, USA) at 75 °C for 10 min (AccuBlock™ Digital Dry Bath, Labnet International Inc., USA) according to the manufacturer’s instructions^45^. The viral DNA aliquot was centrifuged (Spectrafuge™ 24D, Labnet International Inc., USA) at 4000×*g* for 30 min before extraction. The viral DNA was extracted using the Isolate II Genomic DNA Kit (Bioline, UK) according to the manufacturer’s instructions (protocol 9.13). The RNA extraction was performed according to the manufacturer’s instructions using the QIAmp Viral RNA kit (Qiagen, Germany). The RNA was converted to cDNA using the SuperScript First Strand Synthesis System for RT-PCR (Invitrogen, USA) using the random hexamer primers supplied according to the manufacturer’s instructions (Bio-rad T100™ Thermal cycle, Bio-rad Laboratories Inc., USA). The second synthesis (to convert cDNA and ssDNA) was performed using Klenow Fragment (New England Biolabs, USA) (Bio-rad T100™ Thermal cycle, Bio-rad Laboratories Inc., USA). The converted cDNA and ssDNA (along with dsDNA) were amplified with KAPA HiFi polymerase (Roche, Switzerland) and the FR20RV primer as described previously (Bio-rad T100™ Thermal cycle, Bio-rad Laboratories Inc., USA)^46^. All converted cDNA, ssDNA and double-stranded were pooled together.

### Targeted and shotgun metagenomics approach

The targeted metagenomics was performed at Inqaba Biotechnical Industries (Pretoria, South Africa), a commercial NGS service provider. Briefly, the extracted bacterial DNA was amplified by targeting the V1–V3 region of the 16S rRNA gene (using 27F and 518R primers). Paired-end libraries (2 × 300 bp) were prepared using the NEBNext^®^ Ultra™ II DNA library prep kit for Illumina^®^ (New England Biolabs, USA) and sequencing was performed on an Illumina MiSeq instrument (Illumina, USA). After, the targeted approach, a subset of six samples were selected for virome sequencing according to the following criteria: (i) samples should be from both states of disease and (ii) samples should be representative of the diversity in the samples (one for low diversity, one for intermediate diversity and one for high diversity). For shotgun metagenomics of the amplified and pooled virome samples, paired-end libraries (2 × 300 bp) were prepared with the Nextera DNA Flex library preparation kit (Illumina, San Diego, CA, USA) and sequencing performed on an Illumina MiSeq instrument by the National Institute of Communicable Diseases Sequencing Core Facility, South Africa. The fragments of the 16S rRNA sequences were analysed using QIIME2 version 2019.1 (1548866877) and the Greengenes database version 13.8^47–49^. Human DNA was removed from the virome sequences using Bowtie2 Galaxy version 2.3.4.3 using Hg38 genome as a reference genome^50,51^. Thee virome sequences were analysed using Kraken 2 Galaxy version 2.1.1in the Galaxy platform with 2019 virome database^52,53^. The viral sequencing results were compared to the virus-host database (https://www.genome.jp/virushostdb/view/) to determine the host of the viruses identified^54^.

### Statistical analysis and data visualisation

The data was analysed on R using the following packages: (i) Qiime2R version 0.99.21 (to import QIIME2 data), (ii) phyloseq version 1.30.0 (alpha diversity, beta diversity, statistical tests, principal component analysis (PCoA), hierarchical clustering and relative abundance of the taxa), (iii) ggplot2 version 3.3.2 (for the plotting of all graphs), (iv) DESeq2 version 1.26.0 (to determine if there was a log2fold difference) and (v) ALDex2 version 1.20.0^55–59^. A p-value greater than 0.05 was considered significant (for any of the statistical tests unless otherwise specified). The Wilcoxon sum rank test was used as statistical test for the alpha diversity measures.

### Ethics approval and consent to participate

Ethics approval was obtained from the Research Ethics committee, Faculty of Health Sciences, University of Pretoria (REC no: 237/2017). Written informed consent was received from all participants. All methods were performed in accordance with the guidelines and regulations as stipulated by the REC.

### Consent for publication

All authors consent to the publication.

## Results

### Patient demographics

A total of 24 participants were enrolled in the study; 18 males and six females the aged from 50 years old to 82 years old (median age was 60 years old). Only one of the participants was HIV-infected. Participants were distributed across the three hospitals as follows: (i) Hospital A (Tertiary Academic Hospital): 16 participants, (ii) Hospital B (District Hospital): one participant and Hospital C (Private Hospital): seven participants. Eighteen of the participants were in the stable state of disease at the time of sampling and six of the participants were in the exacerbated state of disease at the time of sampling. The clinical characteristics are shown in Table 1.

**Table 1:** Clinical characteristics of COPD participants.

Characteristics	Total patients (n = 24)	Patients in stable state of disease (n = 18)	Patients in exacerbated state of disease (n = 6)
Age (years)	62 .17 ± 7 .34	61 .22 ± 7 .45	65 ± 6 .19
Gender (M:F)	18:6	13:0	2:4
Current smoker	9	8	3
Ex-smoker	11	7	2
Never smoker	4	3	1
Years smoked	27 .8 ± 10 .34	27 .4 ± 10 .03	29 ± 11 .14
Worked in a mine (yes: no)	5:19	4:14	1:5
HIV status (positive: negative)	1:23	1:17	0:6
Previous TB diagnosis (yes: no)	3:21	2:16	1:5
**Hospital recruited from**			
Hospital A	16	12	4
Hospital B	1	0	1
Hospital C	7	6	1

### The sputum microbiome

**Figure 1:**
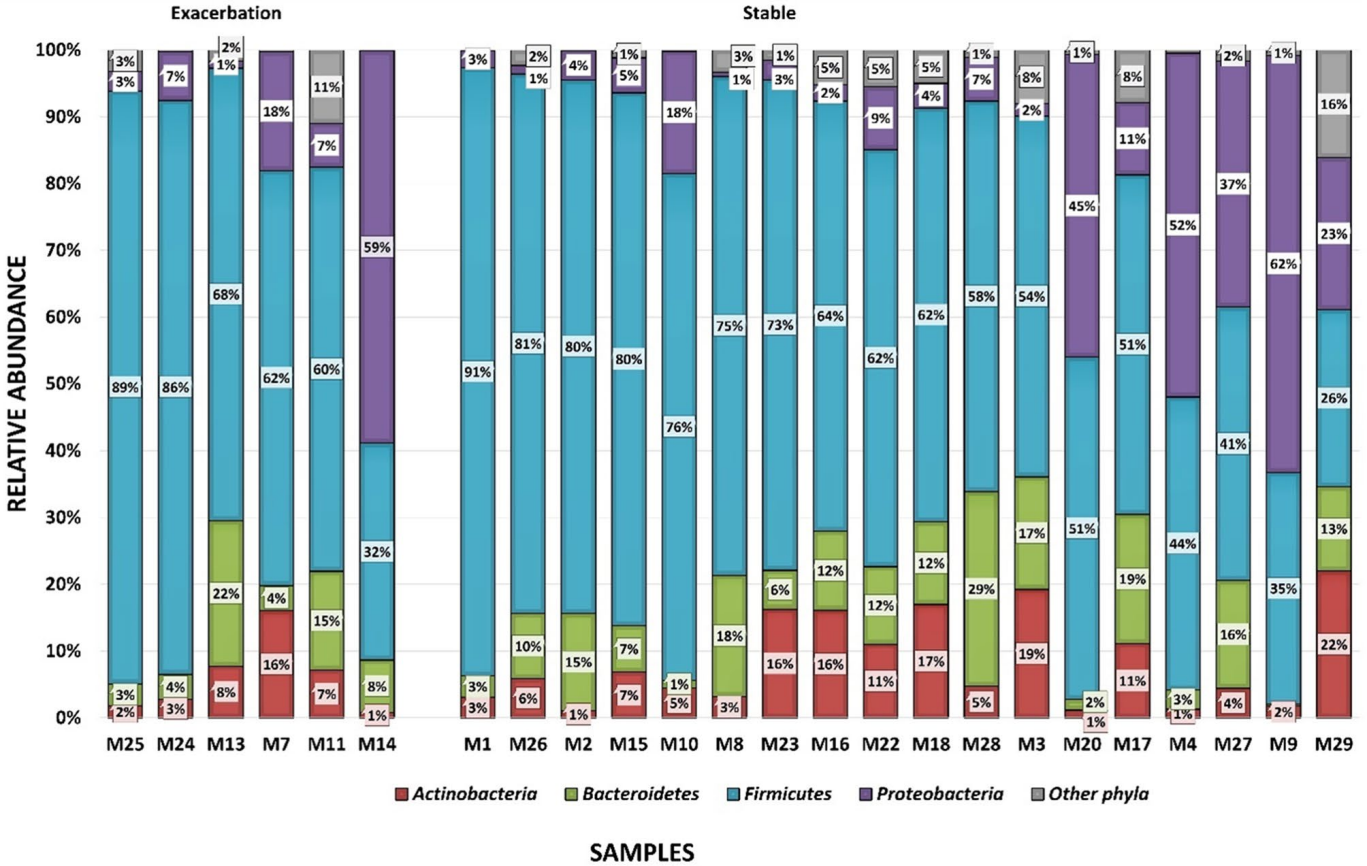
Bar plots showing the relative abundance of the differing phyla by disease state occurring in the sputum microbiome of 24 COPD participants using targeted metagenomics across the different samples. *Firmicutes* are shown in blue, *Proteobacteria* in purple, *Bacteroidetes* in green and *Actinobacteria* in red. The graph is separated into the exacerbated state (n = 6) and stable state (n = 18). The specimens are ordered according to the prevalence of *Firmicutes*.

A total of 631 operational taxonomic units (OTUs) were identified across the 24 samples for the microbiome. These OTUs were divided into 14 phyla, 27 classes, 37 orders, 70 families and 77 genera. Twenty-two percent (140/631) of all OTUs could be classified to a species level. The relative abundance of unclassified species ranged from 32 to 94% between samples. The most abundant phyla identified were *Firmicutes* (ranging from 41 to 91%), *Proteobacteria* (ranging from 3 to 62%), *Bacteroidetes* (ranging from 3 to 22%) and *Actinobacteria* (ranging from 1 to 22%) (Fig. 1).

The most abundant genera were *Streptococcus* (detected in all 24 samples, with abundances ranging from 19 to 82%), *Haemophilu*s (detected in all 24 samples, with abundances ranging from 0.02% to 61%), *Prevotella* (detected in all 24 samples, with abundances ranging from 0.1% to 22%), *Veillonella* (detected in all 24 samples, with abundances ranging from 0.15% to 19%) and *Granulicatella* (detected in all 24 samples, with abundances ranging from 0.12% to 11%).

### Comparison of exacerbation and stable states of disease for the microbiome

The relative abundance of the *Actinobacteria*, *Bacteroidetes*, *Firmicutes*, *Fusobacteria* and *Proteobacteria* phyla differed across the disease states; with a higher abundance of *Firmicutes* (63% in the exacerbated state and 61% in the stable state) and a lower abundance of *Actinobacteria* (5% in the exacerbated state and 5% in the stable state)*, Bacteroidetes* (11% in the exacerbated state and 9% in the stable state) and *Proteobacteria* (19% in the exacerbated state and 17% in the stable state), during the exacerbated state (Figure S1).

**Figure 2:**
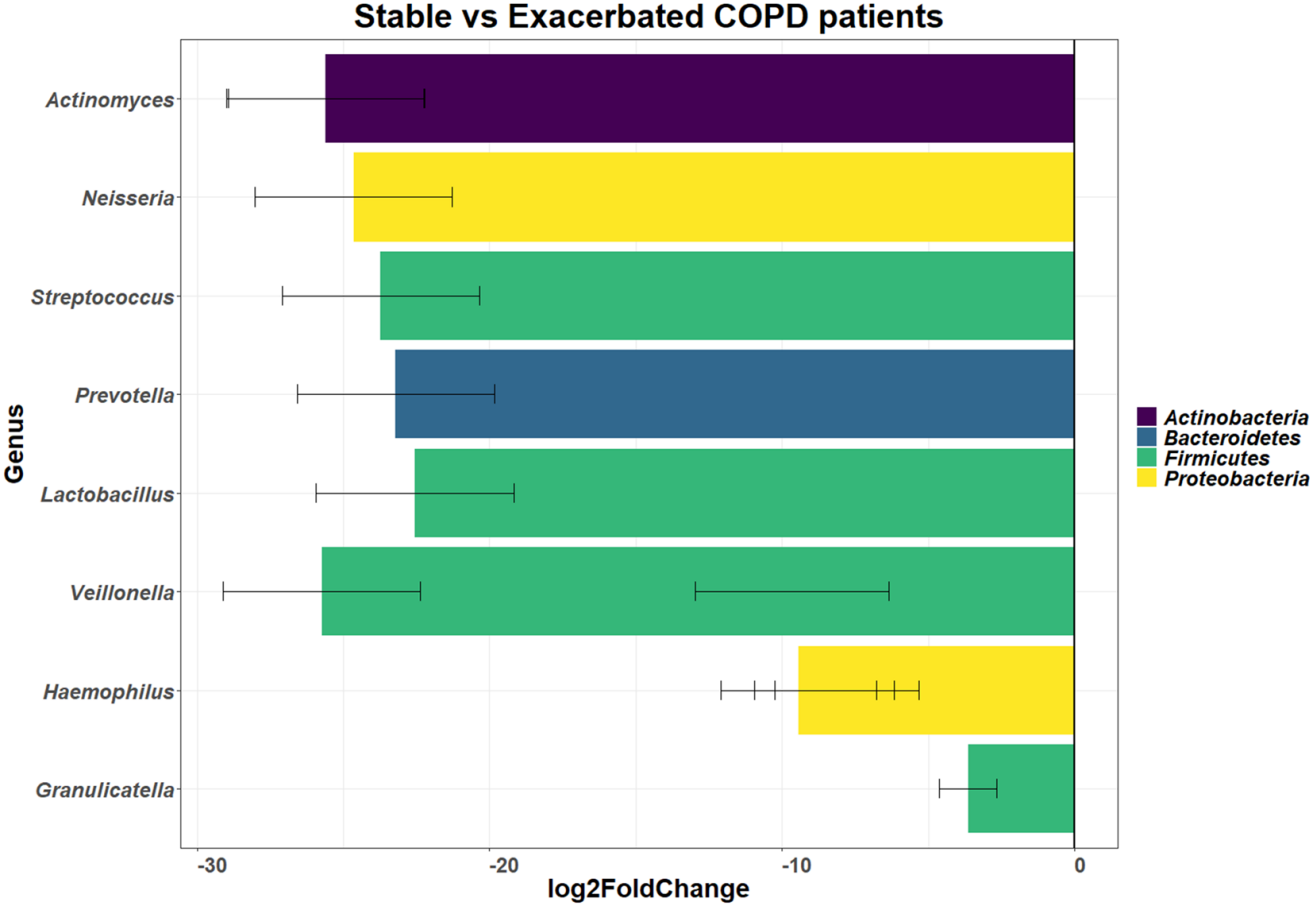
Graph of the DESeq2 analysis showing the log2fold differential abundance of the different genera between the exacerbated state and stable state of disease (n = 24) in the sputum microbiome of COPD participants. Differences were considered significant with the p-value (adjusted for false discovery rate using Benjamini–Hochberg correction) cut-off of 0.2. Log2fold changes greater than zero indicated an increase in the relevant genera, whereas log2fold changes less than zero indicated a decrease in the relevant genera. All genera shown below the zero line had a decreased relative abundance with the stable state of disease i.e. these genera were increased during the exacerbated state of disease. The error bars corresponding to the calculated lfcSE (standard error).

At a genus level (Figure S2), the exacerbated state showed changes in 75 genera; with 49 genera that had a lower relative abundance and 26 genera that had a higher abundance. Key genera that showed lower relative abundance during the exacerbated state included *Porphyromonas* (0.19% in the exacerbated state and 3.92% in the stable state), *Serratia* (0.00% in the exacerbated state and 2.99% in the stable state), *Staphylococcus* (0.00% in the exacerbated state and 1.02% in the stable state) and *Streptococcus* (47.88% in the exacerbated state and 49.61% in the stable state). Genera that showed a higher relative abundance in the exacerbated state included *Granulicatella* (5.30% in the exacerbated state and 3.06% in the stable state), *Haemophilus* (16.82% in the exacerbated state and 11.08% in the stable state), *Prevotella* (10.02% in the exacerbated state and 7.87% in the stable state) and *Veillonella* (6.92% in the exacerbated state and 4.44% in the stable state). Although, the relative abundance differed across the disease state, with DESeq2 analysis and ALDEx2 analysis no significant difference were observed when a false discovery rate (FDR) of 0.05 was used. When an FDR of 0.2 was used, significant differences were observed across the disease states (Fig. 2) for DESeq2 analysis but not for ALDEx2 analysis.

**Figure 3:**
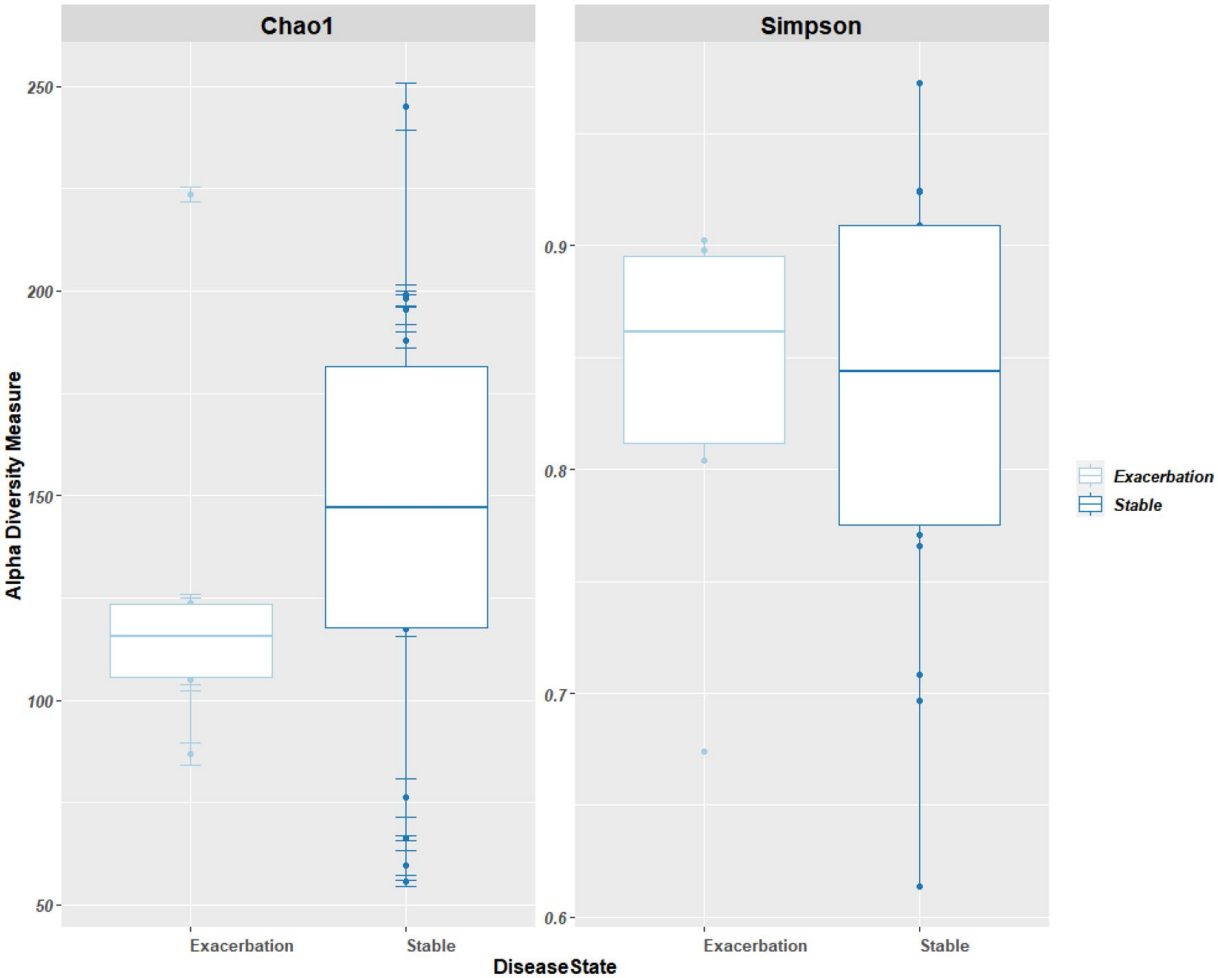
The alpha diversity box-plot of the sputum microbiome compared across the exacerbated state (n = 6) and stable state (n = 18) of COPD using Chao1 and Simpson diversity measures. Each dot on the graph represents a sample. The boxes represent the interquartile range (IQR) and the horizontal line represents the median. The median values for Chao1 diversity measure were as follows: (i) stable state = 147.06 and (ii) exacerbated state = 115.56. The median values for the Simpson diversity measures were as follows: (i) stable state = 0.84 and (ii) exacerbated state = 0.86.

**Figure 4:**
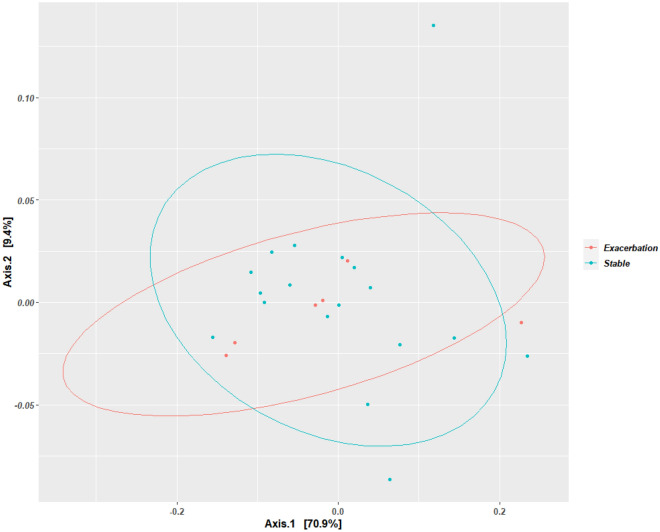
Principal component analysis (PCoA) plot derived using weighted UniFrac diversity measure comparing the different disease states of COPD in the sputum microbiome The ellipses show the different states of disease with the exacerbated state (n = 6) indicated in red and the stable state (n = 18) indicated in blue; with the dots represent in each sample.

There was no significant difference in the alpha-diversity between disease states (Fig. 3) for the microbiome using the Wilcoxon sum rank test for both Chao1 (p-values = 0.58) and Simpson diversity measures (p-value = 0.72). Beta-diversity measures showed no clustering for any of the variables using PCoA and weighted UniFrac (for microbiome) measures (Fig. 4).

### The sputum virome

**Figure 5:**
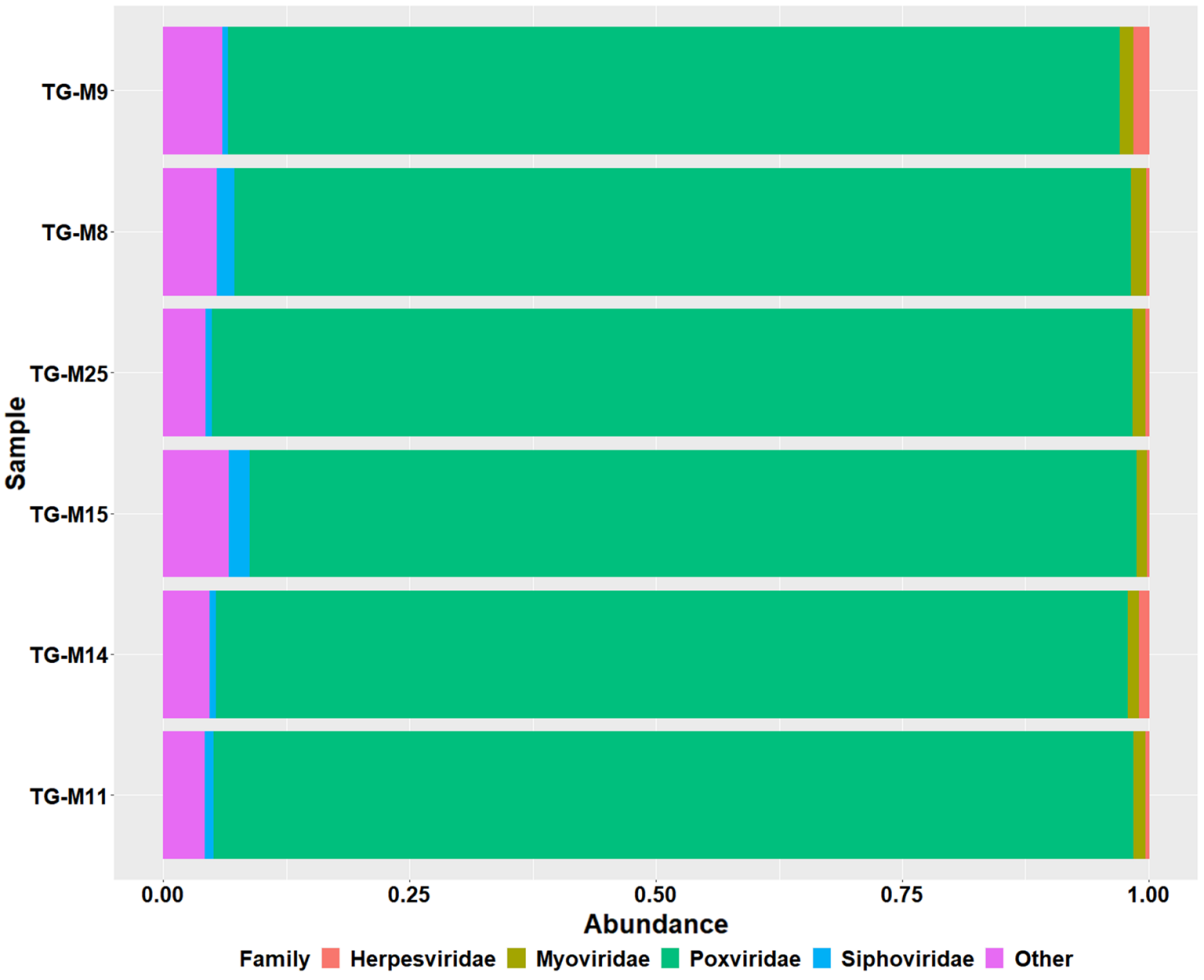
Bar plots showing the most abundant of viruses at a family level; the most prevalent families were as follows: (i) *Poxviridae* (indicated in bright green), (ii) *Siphoviridae* (indicated in blue), (iii) *Myoviridae* (indicated in olive green) and (iv) *Herpesviridae* (indicated in red). The rest of the viruses are grouped together as other (indicated in pink).

A total of 3480 operational taxonomic units (OTUs) were identified across the six samples for the virome. The taxonomic classification identified 16 phyla, 34 classes, 53 orders, 141 families and 826 genera. Most of the OTUs [95% (3306/3480)] could be classified up to a species level. The most abundant family across all samples was the *Poxviridae* family (detected in all six samples, with abundances ranging from 90 to 93%), followed by the bacteriophage families *Myoviridae* (detected in all six samples, with abundances 0.63% to 2.11%) and *Siphoviridae* (detected in all six samples, with abundances 1.08% to 1.55%) (Fig. 5).

The most prevalent species was BeAn 58058, a member of the *Poxviridae* family that was detected in all specimens sent for virome sequencing followed by bacteriophages (associated with both Gram-positive and Gram-negative bacteria). Most of the viruses identified were dsDNA viruses (ranging from 97.23 to 98.15%).

## Discussion

In this study, the composition of the sputum microbiome of COPD participants was investigated and was compared between the different disease states i.e. stable state of disease and exacerbated state of disease. Two phyla predominated, *Firmicutes* and *Proteobacteria*, with *Streptococcus* and *Haemophilus* being the most prevalent genera. However, this study observed no significant differences between the exacerbated and stable states of disease in COPD, in terms of, alpha diversity and beta diversity for the sputum microbiome in COPD. When an FDR of 0.2 was used for DESeq2 analysis, significant differences were observed between the two disease states for the relative abundance. With the virome, a high prevalence of the viruses, BeAn 58058 was observed. In this study, there was difficulty in recruiting HIV-positive individuals with COPD and as a result, only a single HIV-positive participant was recruited in this study.

In both disease states, four phyla dominated: *Firmicutes* (ranging from 41 to 91%), *Proteobacteria* (ranging from 3 to 62%), *Bacteroidetes* (ranging from 3 to 22%) and *Actinobacteria* (ranging from 1 to 22%). This is in agreement with other studies conducted on the lung microbiome in healthy individuals and other lung diseases^60,61^. Even though some of these studies had different patient groups (e.g. asthmatics and smokers), used different specimen types [e.g. bronchoalveolar lavage (BAL)] and used different sequencing technologies (e.g. 454 sequencing), these four phyla were always found to be dominant in the lung microbiome^60–63^. However, the most prevalent phylum has been found to differ between different disease e.g. in severe COPD, *Proteobacteria* is more prevalent whereas in the healthy lung *Firmicutes* is more prevalent^60–65^. This study showed a higher prevalence of *Firmicutes*; previous studies have shown that the microbiome in mild COPD is similar to that of the healthy lung^64,65^. When the stable and exacerbated states of disease were compared in this study a higher abundance of the *Firmicutes* phylum (2% higher in the exacerbated state) and lower abundances of the *Proteobacteria* (2% higher in the exacerbated state), *Actinobacteria* (3% higher in the exacerbated state) and *Bacteroidetes* phyla (2% higher in the exacerbated state) was observed. Previous studies (all conducted using sputum specimens) that have compared the two diseases states in COPD have observed the same trend, where one of the phyla shows an increased prevalence and the other phyla showed a decreased prevalence in the exacerbated state, however, none of these studies reported the percentage increase^66–71^. In most of these studies, *Proteobacteria* increased, however, two studies [Jubinville et al*.* (2018) and Wang et al*.* (2020)], showed an increased prevalence of *Firmicutes* (as observed in this study). These studies had a variety of different sample sizes (ranging from nine participants to 281 participants), were conducted in USA, Europe and China and used different sequencing technologies (454 sequencing, MiSeq sequencing and PhyloChip)^66–71^.

The genera that showed the highest frequency in this study (in both disease states) were: *Granulicatella (Firmicutes)*, *Haemophilus (Proteobacteria)*, *Prevotella (Bacteroidetes), Streptococcus (Firmicutes)* and *Veillonella (Firmicutes)*. Previous studies conducted on the lung microbiome of healthy individuals and COPD patients have observed these genera in high abundances along with *Pseudomonas* and *Porphyromonas*.^72,73^. Most of these studies were conducted using 454 sequencing in the USA or Europe with a variety of different specimens. A study by Wang et al*.* (2016) showed the most similarity to this study with one key difference; the changes in abundance of genera during the exacerbated was different from this study. In this study, *Haemophilus* had a higher abundance [5.7% higher in this study and 3% increase in Wang et al*.* (2016)] whereas *Streptococcus* had a lower abundance [1.7% decrease in this study and 3% decrease in Wang et al*.* (2016)]. These genera i.e. *Granulicatella*, *Haemophilus*, *Prevotella* and *Veillonella* showed significant increase in the relative abundance during the exacerbated state of disease when DESeq2 analysis was used. The differences in abundances of the genera could be attributed to the different study population size (87 individuals in the Wang et al*.* (2016) study vs 24 in this study), the type of country (with United Kingdom (UK) being a developed country and South Africa a developing country) and the difference in the sequencing methodology (MiSeq platform (Illumina, USA) and V1-V3 region for sequencing was used in this study whereas Wang et al*.* (2016) used 454 sequencing (Roche Diagnostics, UK) and the V3-V5 region of 16S rRNA). Geographical location and local environmental conditions, such as air pollution have been shown to affect the lung microbiome and could explain the difference in relative abundance between the two studies^13,74^. Additionally, seasonal variation may play a role in bacteria identified^75^. Most of the exacerbation samples in this study were collected in either autumn or winter. In Pretoria, the dry season is in winter which is in contrast to the United Kingdom, where the dry season generally falls in summer. Additionally, the bacteria that showed a higher prevalence (between 2 to 6% higher) during the exacerbated state of disease, i.e. *Granulicatella*, *Haemophilus*, *Prevotella* and *Veillonella* have been associated with gastrointestinal reflux disease (GERD)^76^. As a result of COPD patients having a common cough, GERD is associated with COPD and is considered a co-morbidity^77^. In fact, GERD has been observed to be a predictor of exacerbations in COPD and implies that a higher prevalence of these bacteria could be used as a potential indicator of COPD exacerbations^77,78^.

In this study, bacterial alpha diversity and beta-diversity analysis showed no difference between disease states. This observation is in agreement with previous COPD studies except for a study by Jubinville et al*.* (2018) who observed a difference in alpha diversity when comparing paired samples i.e. the diversity in the paired samples differed across the disease state with most exacerbated samples showing a higher diversity^66–68,70^. All these studies were conducted in Europe (the UK and Spain) or Northern America (Canada and USA) using sputum specimens, with most studies having less than 30 participants and having used 454 sequencing. The only difference between these studies and the study by Jubinville et al*.* (2018) was the diversity measure used; most of the other studies used the Shannon index (often combined with Chao1 and Faith PD diversity measure), whereas Jubinville et al*.* (2018) used the Simpson index. Unlike, the Shannon index, the Simpson index is more affected by the relative abundances (i.e. evenness) of the species in a sample; this suggests that during the exacerbated state of disease, the abundances of species/OTUs changes but not the number of species/OTUs (richness)^79^.

In this study, the most prevalent viral family was *Poxviridae* followed by *Siphoviridae* and *Myoviridae*. When compared to the only two other studies that have focused on the COPD lung virome, this study differed in the relative abundance of the key families^40,41,87^. The study by Garcia-Nunez et al*.* (2018) used sputum specimens (n = 10) from paired stable and exacerbated patients (n = 5) in Spain. The study by van Rijn et al*.* (2019) used nasopharyngeal swabs (n = 88) collected from exacerbated patients between 2006 and 2010 and was conducted in Norway. The most prevalent viral families in these studies were *Anelloviridae* (negative sense DNA virus with no known pathogenicity in humans) and *Siphoviridae* (double-stranded DNA bacteriophages that have been found in the lung virome of CF patients as well as in the gastrointestinal tract virome and the oral virome^40,41,80–85^. These bacteriophages i.e. *Siphoviridae* and *Myoviridae* may act as reservoirs for antibiotic resistance genes (contain antibiotic resistance genes in their genomes), mobile genetic elements and may contain virulence genes and other genes that affect bacterial metabolic pathways^35,86^.

A high abundance of *Poxviridae* was observed in this study, particularly the BeAn 58,085 virus (BAV). *Poxviridae* is a family of complex, double-stranded DNA (dsDNA) viruses that are often zoonotic and are known to cause skin lesion, with the most well-known virus being *variola virus*, the causative agent for smallpox (has been eradicated)^87^. Only two other virome studies, one that studied fluid in the human body (conducted in Spain) and one that studied ocular adnexa (conducted in Denmark on samples collected between 2005 and 2014) detected the BeAn 58058 virus in humans^88,89^. This virus (BeAn 58058) was originally isolated from rodents (*Oryzomys* sp.) in Brazil in 1963^90^. According to the viral-host database, the only known host for the BeAn 58058 virus is the *Oryzomys* sp., however, other *Poxviridae* have been known to infect a wide variety of hosts including humans^54^. The BeAn 58085 virus is considered a variant of the *Vaccinia virus*, a close relative of the smallpox virus that was used as a vaccine vector for smallpox until 1970^91,92^. There are three possible explanations for the high abundance of BeAn 58058 virus detected in this study. The first theory is that the BeAn 58058 virus is an ancient virus that over time has incorporated as part of the human genome; the theory is supported by (i) A study by Mollerup et al*.* (2019) conducted on the virome of the ocular adnexa, which showed that viral reads (i.e. the BeAn 58058 virus) identified had high sequence homology to sequences of human origin, (ii) A study that was conducted on the human genome (studying structural variants) identified the BeAn 58058 virus as part of the genome and iii) *Poxviridae* re dsDNA viruses and can easily integrate into the double-stranded human genome^93^. The second theory is that BeAn 5808 is a DNA artefact of the smallpox vaccine (which was a live attenuated vaccine) received years earlier; evidence supporting this theory includes the following: (i) the study population in this study were all over the age of 50 years and would have received the smallpox vaccine before the vaccination programme for the smallpox virus was terminated in South Africa (in 1970) and (ii) the *Vaccinia virus*, which was used for the smallpox vaccine showed high homology with the BeAn 58058 virus^91,92,94^. The third theory is that the participants in this study encountered an environmental exposure from which the virus was contracted, e.g. rats and its similarity to the *cotia virus*, which can infect human cells^95^. The fourth theory is that the BeAn 5808 is a contaminant (i.e. a sequence not truly in the sample) from the extraction kit, from animal cells, reagents used or even from a previous sequencing run^96,97^. Further analysis of the lung virome, as well as the human genome of healthy individuals (i.e. not suffering from any lung disease) across different geographical regions and age groups, should provide insight into this in the future.

This study had several limitations. First, this study had a small population size and did not have paired samples for the different disease states. Second, a sputum specimen was chosen for this study (instead of BAL, which has been used by most studies on the COPD microbiome) as it is the most patient-friendly method i.e. is non-invasive^98^. The sputum microbiome has a mixture of the microbiomes from both the upper respiratory tract and the lower respiratory tract^98–101^. Additionally, sputum specimens have higher bacterial loads and are better for longitudinal studies (as these specimens are non-invasive)^99^. Third, as only a single HIV participant could be recruited into this study, no comparison between HIV positive individuals and HIV negative individuals could be performed for the sputum microbiome in COPD patients; this aspect therefore requires further research. Lastly, no controls were included in the study; the lack of negative controls for the extraction procedure (conducted in a Biosafety level 2 cabinet with DNase away and RNase away) means that the laboratory contamination from extraction reagents, from a previous sequencing run, etc. cannot be ruled out^96,97,101^. However, a strength of this study was that it provided a good pilot overview of the sputum microbiome and the sputum virome of the COPD lung in a South African setting. A diverse microbiome was observed in this study in both the stable and the exacerbated states of disease; with *Proteobacteria* predominating in the exacerbated state of disease. Conversely, the virome (studied both DNA and RNA viruses) was dominated by a single virus, the BeAn 58058 virus (a dsDNA virus). Most viruses found previously found in respiratory tract were shown to be RNA viruses, such as Influenza viruses, however, most shotgun metagenomics approaches favour DNA viruses, such as members of the *Siphoviridae*. As result members of the *Siphoviridae* family and other DNA viruses, such as BeAn 58058 dominate the lung virome. However, the origins of the BeAn 58085 virus and its possible clinical relevance is unknown. Future studies into the virome would require further investigation into this virus by studying the lung virome in healthy individuals and other lung diseases in the South African and international context. Future studies into the COPD lung microbiome should include longitudinal studies that compared the stable and exacerbated states of disease over several time points in the same individuals.

## Conclusions

This study is among the first to report lung microbiome composition in COPD patients from Africa. No statistically significant differences in the microbiome of COPD patients during the different states of disease were observed in this study. However, this study did note differences in the frequencies of key phyla and genera when compared to other studies from Europe and the USA. However, the reason for this differing microbial profile is unknown and warrants further research. In the virome, a high frequency of the BeAn 58058 virus was observed in the six samples; the explanation for this observation is unclear. To conclude, the sputum microbiome in South African COPD patients is diverse, regardless of the disease state, while the sputum virome warrants further research.

## Supplementary Information

Supplementary Information.

## Data Availability

The sequencing data from this study is available in the NCBI Sequence Read Archive (SRA) database (https://www.ncbi.nlm.nih.gov/sra) Bioproject PRJNA683885 (Accession numbers SAMN17041381 to SAMN17041404 and SAMN17065738 to SAMN17065743). The scripts used in R and in QIIME2 were added to a Github respiratory at https://github.com/tgmahomed/COPDMicrobiome.

## Abbreviations

16S rRNA: 16S ribosomal ribonucleic acid
cDNA: Complementary deoxyribonucleic acid
COPD: Chronic obstructive pulmonary disease
DNA: Deoxyribonucleic acid
DNase: Deoxyribonuclease
dsDNA: Double stranded deoxyribonucleic acid
dsRNA: Double stranded ribonucleic acid
DTT: Dithiothreitol
FEV1%: Percentage of the forced vital capacity
HIV: Human immunodeficiency virus
IQR: Interquartile range
N/A: Not available
NGS: Next generation sequencing
NICD: National Institute for Communicable Diseases of South Africa
OTUs: Operational taxonomic units
PCoA: Principal component analysis
QIIME2: Quantitative insights into microbial ecology 2
REC: Research ethics committee
RNA: Ribonucleic acid
rRNA: Ribosomal ribonucleic acid
RT-PCR: Reverse transcriptase polymerase chain reaction
ssDNA: Single stranded deoxyribonucleic acid
ssRNA: Single stranded ribonucleic acid
UK: United Kingdom
USA: United States of America
