# Supplementary material for: Lung microbiome of stable and exacerbated COPD patients in Tshwane, South Africa
Source: Sci Rep. 2021 Oct 5;11:19758. doi: 10.1038/s41598-021-99127-w (PMC8492659; doi:10.1038/s41598-021-99127-w)
Supplement: Supplementary file 1 — Supplementary Information. [file 41598_2021_99127_MOESM1_ESM.docx]

**Lung microbiome of stable and exacerbated COPD patients in Tshwane, South Africa**

**Goolam Mahomed T^1^, Peters RPH^1,2^, Allam M^3^, Ismail A^3^, Mtshali S^3^, Goolam Mahomed A^4^, Ueckermann V^5^, Kock MM^1,6^, and Ehlers MM^1,6*^**

1. **Department of Medical Microbiology, University of Pretoria**
2. **Foundation for Professional Development, Research Unit, East London, South Africa**
3. **National Institute for Communicable Diseases, of the National Health Laboratory Service, Johannesburg, South Africa**
4. **Louis Pasteur Private Hospital**
5. **Department of Internal Medicine, University of Pretoria**
6. **Department of Medical Microbiology, Tshwane Academic Division, National Health Laboratory Service**

*****Corresponding author: marthie.ehlers@up.ac.za

**Supplementary Materials and Results**

Table S1: Inclusion and exclusion criteria for COPD patients in this study

| **Stable state** | |
| --- | --- |
| **Inclusion criteria** | **Exclusion criteria** |
| HIV patients on antiviral therapy (ART) | Active tuberculosis infection (receiving treatment) |
| Over 40 years of age | Receiving immunosuppressants |
| Able to provide informed consent | Cancer |
|  | Lung surgery within the last six months |
|  | Unable to answer questionnaire (CDQ) |
|  | Antibiotics within last month |
| **Exacerbated state** | |
| **Inclusion criteria** | **Exclusion criteria** |
| HIV patients on antiviral therapy (ART) | Active tuberculosis infection (receiving treatment) |
| Over 40 years of age | Receiving immunosuppressants |
| Able to provide informed consent | Cancer |
| Increased/worsening of respiratory symptoms 48 h prior to visit | Lung surgery within the last six months |
|  | Unable to answer questionnaire (CDQ) |
|  | Unable to give informed consent |
|  | Antibiotics therapy 24 h prior to admission |
|  | Antibiotic therapy administered for more than 12 h after admission |

FEV1% - The ratio of FEV_1_ (forced expiratory volume in 1 second, the amount of air that can be blown out after a second) to FVC (forced vital capacity, the amount of air that can be blown out after a full inspiration)

# **Prevalence of different species**

The most abundant species in the 22% of the OTUs that could be classified to species level were: i) *Haemophilus influenzae* (detected in 21/24 samples, with abundance ranging from 0.01% to 61%), ii) *Haemophilus parainfluenzae* (detected in 22/24 samples, with abundance ranging from 0.01% to 16%), *Prevotella melaninogenica* (detected in all 24 samples, with abundance ranging from 0.08% to 15%), *Veillonella dispar* (detected in 21/24 samples, with abundance ranging from 0.02% to 9%) and *Veillonella parvula* (detected in 23/24 samples, with abundance ranging from 0.07% to 9%). Additionally, sample M20 showed a high abundance of *Serratia marcescens* (41%), sample M4 showed a high abundance of *Pseudomonas* spp. (49%) (was not classified to a species level) and sample M26 showed a high abundance of *Staphylococcus aureus* (13%).


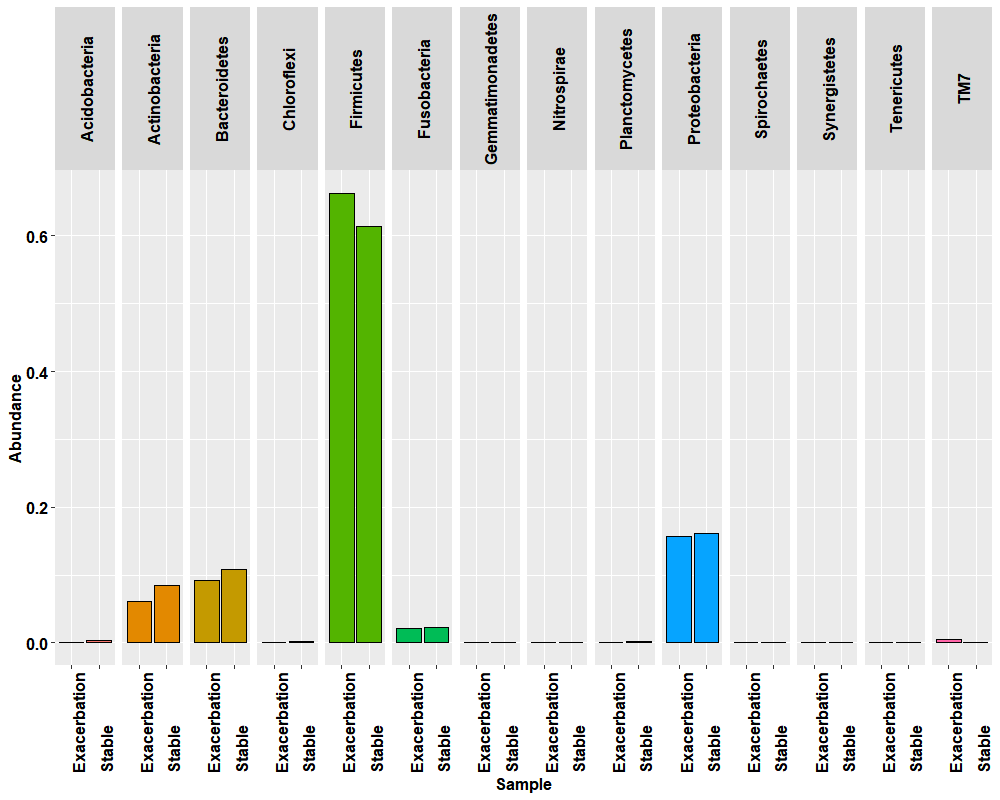


Figure S1: Bar plots showing the relative abundance of the different phyla in the sputum microbiome of COPD participants as determined by targeted metagenomics compared across exacerbated state (n=6) and stable state (n=18).


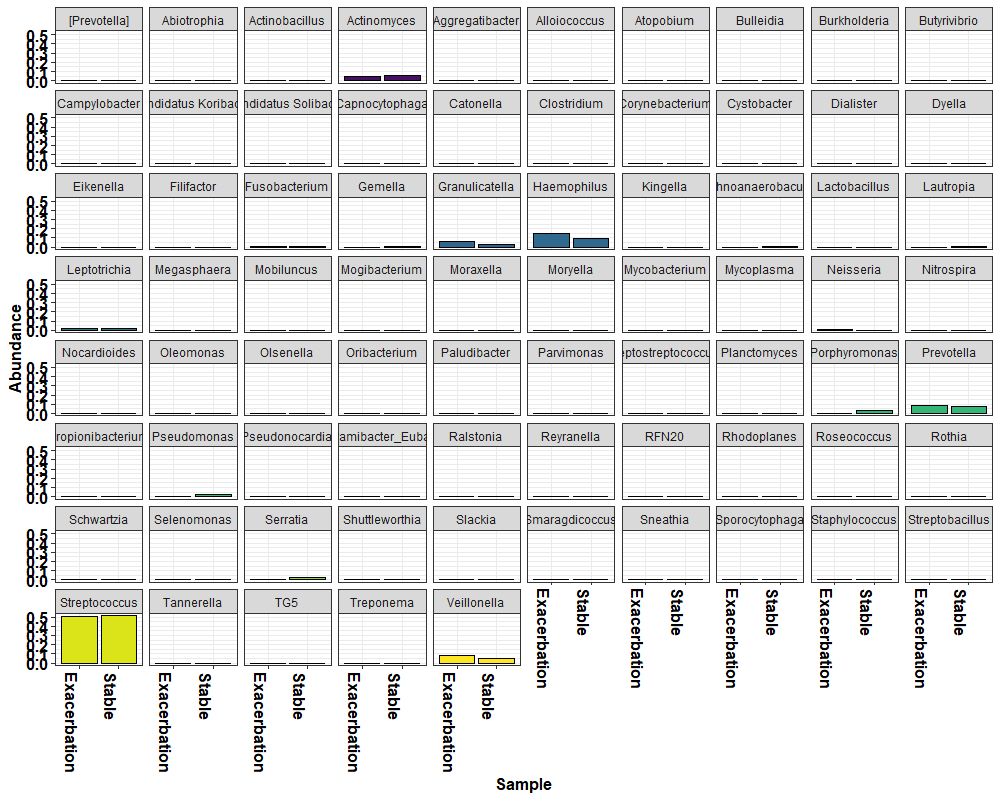


Figure S2: Bar plots showing the relative abundance of the genera in the sputum microbiome of COPD participants by disease state.


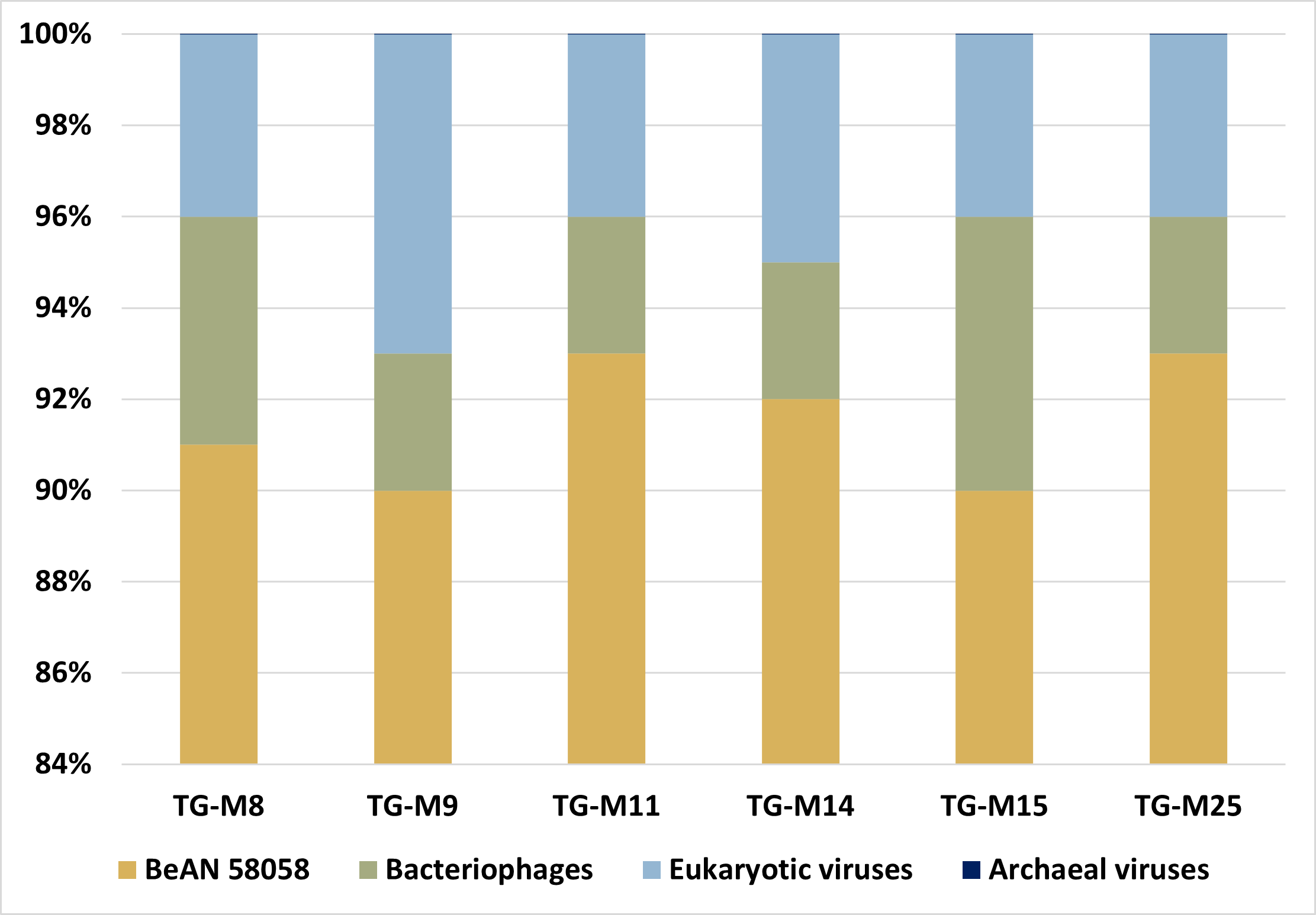


Figure S3: Bar plot showing the distribution of viruses (obtained from shotgun metagenomic sequencing using the Kraken 2 virome database) across the different samples (n=6) of the sputum virome of COPD participants based on their hosts.
